# Genetically Predicted Blood Pressure Across the Lifespan: Differential Effects of Mean and Pulse Pressure on Stroke Risk

**DOI:** 10.1161/HYPERTENSIONAHA.120.15136

**Published:** 2020-07-06

**Authors:** Marios K. Georgakis, Dipender Gill, Rainer Malik, Athanase D. Protogerou, Alastair J.S. Webb, Martin Dichgans

**Affiliations:** 1 From the Institute for Stroke and Dementia Research, University Hospital, LMU Munich, Germany (M.K.G., R.M., M.D.); 2 Department of Biostatistics and Epidemiology, School of Public Health, Imperial College London, United Kingdom (D.G.); 3 Cardiovascular Prevention and Research Unit, Department of Pathophysiology, National and Kapodistrian University of Athens, Greece (A.D.P.); 4 Centre for Prevention of Stroke and Dementia, Department of Clinical Neurosciences, University of Oxford, United Kingdom (A.J.S.W.); 5 Munich Cluster for Systems Neurology (SyNergy), Munich, Germany (M.D.); 6 German Centre for Neurodegenerative Diseases (DZNE), Munich, Germany (M.D.).

**Keywords:** arterial pressure, blood pressure, hypertension, Mendelian randomization analysis

## Abstract

Hypertension is the leading risk factor for stroke. Yet, it remains unknown whether blood pressure pulsatility (pulse pressure [PP]) causally affects stroke risk independently of the steady pressure component (mean arterial pressure [MAP]). It is further unknown how the effects of MAP and PP on stroke risk vary with age and stroke cause. Using data from UK Biobank (N=408 228; 38–71 years), we selected genetic variants as instruments for MAP and PP at age ≤55 and >55 years and across age deciles. We applied multivariable Mendelian randomization analyses to explore associations with ischemic stroke, intracerebral hemorrhage, and their subtypes. Higher genetically predicted MAP was associated with higher risk of ischemic stroke and intracerebral hemorrhage across the examined age spectrum. Independent of MAP, higher genetically predicted PP only at age >55 years was further associated with higher risk of ischemic stroke (odds ratio per-SD-increment, 1.23 [95% CI, 1.13–1.34]). Among subtypes, the effect of genetically predicted MAP on large artery stroke was attenuated, whereas the effect of genetically predicted PP was augmented with increasing age. Genetically predicted MAP, but not PP, was associated with small vessel stroke and deep intracerebral hemorrhage homogeneously across age deciles. Neither genetically predicted MAP nor PP were associated with lobar intracerebral hemorrhage. Beyond an effect of high MAP at any age on ischemic and hemorrhagic stroke, our results support an independent causal effect of high PP at older ages on large artery stroke. This finding warrants further investigation for the development of stroke preventive strategies targeting pulsatility in later life.

## Abstract 2

Supplemental Digital Content is available in the text.

Stroke ranks as the second most common cause of death and the commonest cause of disability worldwide.^1,2^ Hypertension is the leading risk factor for both ischemic and hemorrhagic stroke (intracerebral hemorrhage [ICH])^3,4^ and accounts for half of the population attributable risk worldwide.^3,4^ Blood pressure lowering lies at the core of primary and secondary stroke prevention strategies.^5,6^ We recently showed that high genetically predicted systolic blood pressure (SBP) and diastolic blood pressure (DBP) are associated with all major ischemic stroke subtypes (large artery, cardioembolic, small vessel stroke), as well as with deep, but not lobar ICH.^7^ While these results support a causal association of elevated blood pressure with the main stroke subtypes, it remains unclear how these associations vary across the lifespan,^8,9^ given the hemodynamic changes that take place as a result of aging.^10^

With older age the large arteries become stiffer, thus leading to an increase in blood pressure pulsatility (pulse pressure [PP]), defined as the difference between SBP and DBP.^10^ This is associated with a greater transmission of pulsatile flow into the distal circulation and can lead to damage of end-organ vascular beds including the cerebral circulation.^11,12^ Indeed, in observational studies, higher PP has been associated with risk of cardiovascular disease, including stroke, beyond mean arterial pressure (MAP).^13–17^ However, observational studies assess only a limited period of the natural history of cardiovascular disease, thus making it difficult to disentangle the dynamic interactions between MAP and PP and their associations with cardiovascular disease. For example, while increasing PP might accelerate vascular pathologies, the reverse might also be true, as, for example, atherosclerotic plaques lead to local arterial stiffening and higher PP.^18^ Furthermore, there are no studies that sytematically explored the effects of MAP and PP across different age strata on stroke risk and how effects vary between etiologically defined stroke subtypes. Given the differential efficacy of different antihypertensive drug classes for lowering MAP and PP,^19,20^ it is important to determine these associations to optimize blood pressure lowering approaches for stroke prevention.

By using genetic variants randomly allocated at conception as instrumental variables for traits of interest, Mendelian randomization (MR) overcomes key limitations of observational studies, assesses lifelong exposures to risk factors, and can clarify potential causal associations.^21^ By drawing on genome-wide association studies (GWAS) with detailed phenotyping of stroke cases, MR further enables the exploration of etiological stroke subtypes. Here, we leveraged data from large-scale GWASs and the UK Biobank (UKB) and performed MR analyses to explore the independent effects of genetically predicted MAP and PP across different age strata on risk of ischemic stroke, ICH, and their main etiological subtypes in subjects aged 38 to 71 years.

## Methods

### Access to Data and Ethical Approval

The analyses for this study were based on publicly available summary statistics. The original studies have received institutional review board approval as appropriate. Data from the UKB can be accessed upon submission of a research proposal (application 2532 for the current project). The summary data for the current analysis are available in the main article and in the Data Supplement. This MR study was performed in accordance with the STROBE-MR criteria.^22^ All statistical analyses were undertaken using R (v3.5.0; The R Foundation for Statistical Computing).

### GWASs for Age-Stratified Blood Pressure Traits in UKB

Data sources are detailed in Table 1. We performed GWAS for MAP and PP in the UKB population after stratifying the sample to individuals ≤55 and >55 years of age. We used the mean SBP and DBP from the 2 brachial measurements (automated or manual) performed at the baseline assessment and added 15 and 10 mm Hg to these values, respectively for individuals under treatment with antihypertensive agents.^23^ PP was calculated as (SBP−DBP), whereas MAP was calculated as (DBP+[1/3]×PP). In the pooled sample, mean MAP and PP were 103.2 (SD: 11.9) mm Hg and 56.7 (SD: 13.6) mm Hg, respectively. We selected a threshold of 55 years of age, because this is the median of the age spectrum covered by UKB, and this represents approximately the turning point, when the association between DBP and age is reversed, due to increasing arterials stiffness.^10,24^ Related individuals up to second-degree were excluded (relatedness coefficient <0.0884).^25^ We performed linear regression using age, sex, the first 20 principal components, genotyping chip, and assessment center as covariates.

**Table 1.**
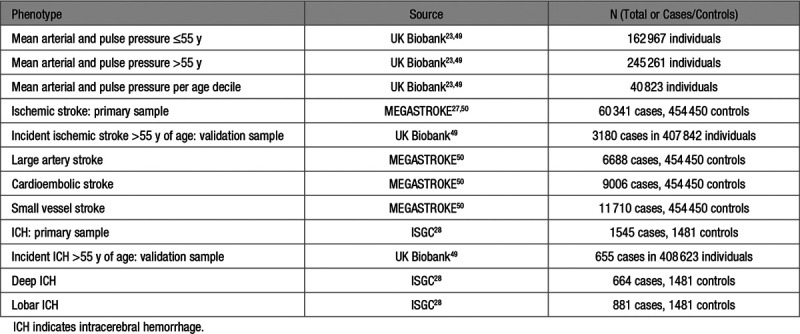
Data Sources That Were Used in the Analyses for the Current Mendelian Randomization Study

### Selection of Genetic Instruments

We identified genetic instruments for MAP and PP at age ≤55 and >55 years as single-nucleotide polymorphisms (SNPs) associated with MAP or PP at genome-wide significance level (*P*<5×10^−8^) and clumped based on the European 1000 Genomes panel to *r*^2^<0.01. We identified a total of 486 SNPs fulfilling these criteria, as detailed in Table I in the Data Supplement. We estimated the proportion of variance in MAP and PP in the 2 age groups explained by genetic variants associated with these phenotypes at *P*<5×10^−8^ using standard formulae.^26^

### Associations With Outcomes

#### Primary Sample

The primary outcomes for our analyses were ischemic stroke and its Trial of Org 10172 in Acute Stroke Treatment-defined etiological subtypes (large artery, cardioembolic, small vessel stroke), as well as ICH and its subtypes defined by hemorrhage location (deep and lobar). Genetic association estimates for ischemic stroke, and its subtypes were obtained from the MEGASTROKE multi-ethnic GWAS meta-analysis of 60 341 ischemic stroke cases (6688 large artery, 9006 cardioembolic, and 11 710 small vessel stroke cases) and 454 450 controls.^27^ For ICH, we used summary statistics from the International Stroke Genetics Consortium GWAS meta-analysis including 1545 cases (664 lobar, 881 deep) and 1481 controls.^28^

#### Validation Sample

MEGASTROKE and ISGC also include individuals with ischemic stroke or ICH occurring at ≤55 years of age (although they comprise in both data sets <10% of the samples). To validate the temporal trends between genetically predicted blood pressure at age ≤55 and >55 years with the risk of ischemic stroke and ICH, we further performed additional analyses in the UKB, restricted to incident first-ever events (occurring after the baseline blood pressure measurements) in individuals >55 years of age. These analyses included 3180 incident ischemic stroke cases among 407 842 individuals and 655 incident ICH events among 408 623 individuals. We explored the genetic effects of the identified instruments using Cox regression analyses to also consider in the analyses the time interval between blood pressure measurement and the occurrence of the incident events.

### Statistics

#### Univariable Analyses

We first performed univariable MR analyses restricted to the instruments specific for genetically predicted MAP and PP at age ≤55 or >55 years. The MR effect estimates were derived from multiplicative random-effects inverse-variance weighted MR analyses.^29^ Statistical significance was set at *P*<0.05. Because inverse-variance weighted is based on the assumption that the genetic instruments do not exert directional pleiotropy, to explore the robustness of our findings to the use of potentially pleiotropic variants, we further applied the weighted median estimator and the contamination mixture model. The weighted median estimator provides consistent estimates as long as at least half of the variants used in the MR analysis are valid.^30^ The contamination mixture model constructs a likelihood function of the individual estimates and under the assumption that the estimates of the valid instruments would follow a distribution centered around the causal effect and any invalid instruments would follow a distribution around zero, it calculates MR estimates that would maximize this likelihood.^31^ The contamination mixture model assumes that some of the genetic variants used are valid instruments.^31^

#### Multivariable Analyses

Given the high genetic and phenotypic correlation between MAP and PP, we performed summary-data multivariable MR to disentangle their independent associations with ischemic stroke, ICH, and their subtypes.^32^ Multivariable MR takes into account the effects of the genetic instruments on both exposure phenotypes (MAP and PP) and thus allows us to disentangle the direct effects of the 2 phenotypes on the outcomes of interest, despite the presence of genetic overlap between them.^32^ For analyses for the effects of MAP and PP at age ≤55 years, we used 220 genetic variants associated with MAP or PP at *P*<5×10^−8^ in this age group, whereas for MAP and PP at age >55 years, we respectively used 450 genetic variants associated with MAP or PP in this age group. Statistical significance was set at *P*<0.05. To deal with the imbalance between the number of genetic instruments for MAP and PP, which could explain differences in the derived estimates, in sensitivity analyses we randomly selected 100 SNPs associated with MAP plus 100 SNPs associated with PP in each age group and re-performed the multivariable analyses 1000 times.^33^

#### Analyses Per Age Decile

To explore associations of genetically predicted MAP and PP across the entire age spectrum, we used all the 486 SNPs associated with MAP or PP at age ≤55 or >55 years and explored associations with MAP and PP across age deciles of the UKB at baseline. We then used these SNPs to perform multivariable MR analyses for the effects of both MAP and PP on risk of the examined outcomes at every decile. To explore trends of effect modification across the age spectrum, we then performed linear meta-regression analyses between the derived effect estimates for every decile and the respective median age per decile. Statistical significance was set at *P*<0.05.

## Results

The baseline characteristics of individuals ≤55 or >55 years of age in the UKB are available in Table 2. A total of 486 independent genetic variants were identified to be associated with MAP or PP at age ≤55 or >55 years (Table S1). Of those, 133 and 100 variants were associated with MAP and PP at age ≤55 years and explained 3.6% and 1.6% of the variance, respectively. Furthermore, 197 and 249 variants were specifically associated with MAP and PP at age >55 years explaining 3.3% and 5.7% of the variance, respectively. There was some overlap between the genetic variants associated with MAP and PP at age ≤55 and >55 years (Table S1). Specifically, 28 of the 205 variants (14%) associated with MAP or PP at age ≤55 years (*P*<5×10^−8^) showed significant associations with both phenotypes. In the older age stratum (>55 years), 47 of the 399 variants (12%) associated with MAP or PP showed significant associations with both phenotypes (Figure S1). In the univariable inverse-variance weighted MR analyses, we found both higher genetically predicted MAP and PP at both ages ≤55 and >55 years to be associated with higher risk of ischemic stroke (Table S2). Regarding ICH, we found significant associations with higher genetically predicted MAP at age ≤55 and >55 years, as well as with higher genetically predicted PP at age >55 years. These results were stable in sensitivity alternative MR analyses (weighted-median, contamination-mixture; Table S2).

**Table 2.**
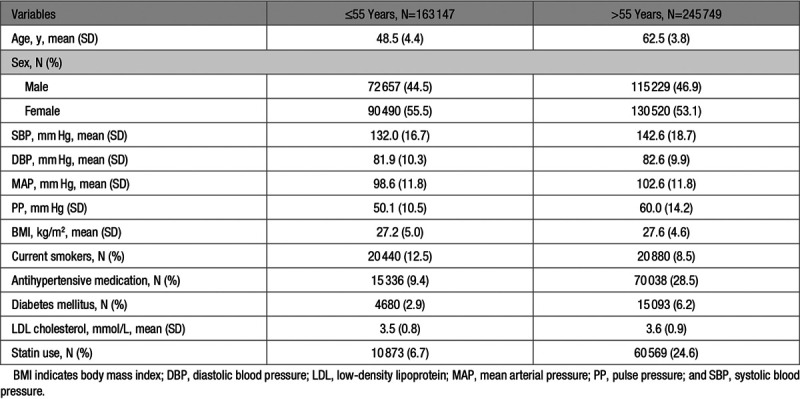
Baseline Characteristics of Participants in the UK Biobank Stratified by Age (≤55 y and >55 y)

**Figure 1.**
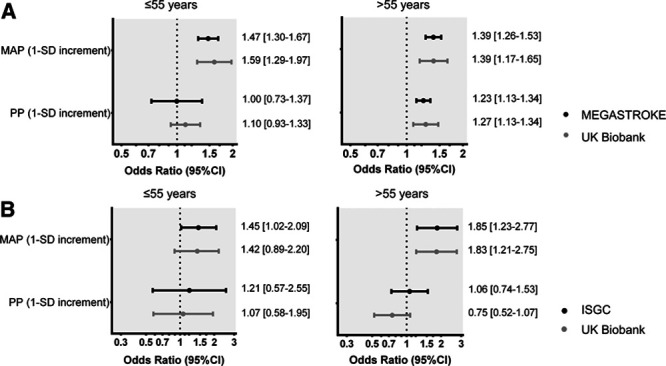
**Associations of genetically predicted mean arterial pressure (MAP) and pulse pressure (PP) at age ≤55 and >55 y with risk of (A) ischemic stroke and (B) intracerebral hemorrhage.** Effect sizes are derived from multivariable Mendelian randomization analyses adjusting for both genetically predicted MAP and PP in MEGASTROKE (primary sample, 60 341 cases, 454 450 controls) and the UK Biobank (validation sample, 3760 cases among 408 623 individuals). The analysis of the UK Biobank is based on incident events that occurred in individuals >55 y of age after the blood pressure measurements.

To disentangle the independent effects of genetically predicted MAP and PP, we performed multivariable MR analyses. In the primary MEGASTROKE sample, higher genetically predicted MAP at either ≤55 or >55 years of age was associated with higher risk of ischemic stroke independently of PP (odds ratio [OR] per 1 SD increment, 1.47 [95% CI, 1.30–1.67] at ≤55 years; OR, 1.39 [1.26–1.53] at >55 years). On the contrary, only genetically predicted PP at age >55 years showed an association with ischemic stroke beyond genetically predicted MAP (OR, 1.11 [0.93–1.33] at ≤55 years; OR, 1.23 [1.13–1.34] at >55 years; Figure 1A). With regard to ICH, there were significant associations between higher genetically predicted MAP at both ≤55 and >55 years of age with a higher risk (OR per 1 SD increment, 1.45 [95% CI, 1.02–2.09] at ≤55 years; OR, 1.85 [1.23–2.77] at >55 years), whereas genetically predicted PP at either age stratum showed no significant independent association (Figure 1B). The results for both ischemic stroke and ICH were consistent in the validation sample of the UKB (sensitivity analysis), when restricting ischemic stroke cases to first-ever incident events occurring after 55 years of age (Figure 1). The results were also consistent in sensitivity analyses randomly selecting 100 genetic variants associated with MAP and 100 variants associated with PP for the young and the old age strata. This sensitivity was done to account for the imbalance in the number of instruments and variance explained for MAP and PP across the age groups (Figure S2).

**Figure 2.**
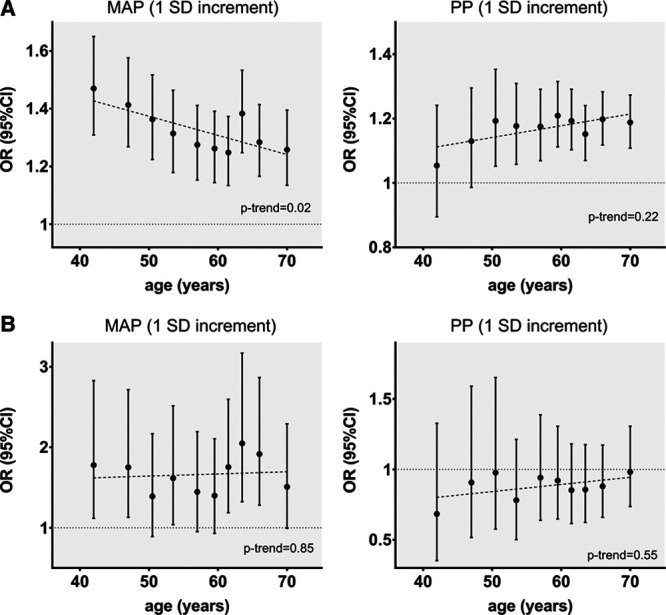
**Associations of genetically predicted mean arterial pressure (MAP) and pulse pressure (PP) across age deciles with risk of (A) ischemic stroke and (B) intracerebral hemorrhage.** Effect sizes are derived from multivariable Mendelian randomization analyses adjusting for both genetically predicted MAP and PP in every decile. The analyses are based on the MEGASTROKE sample for ischemic stroke and on the ISGC sample for intracerebral hemorrhage. Trends across age were explored with linear meta-regression analyses. OR indicates odds ratio.

To explore the modifying effects of age on the associations between genetically predicted MAP and PP on risk of ischemic stroke and ICH across the entire age spectrum of the UKB population, we next explored associations across age deciles (Figure 2 and Table S3). Genetically predicted MAP was significantly associated with the risk of ischemic stroke across all age deciles, but there was a trend for an attenuation of the effect with increasing age (*P*=0.02; Figure 2A). We found no significant effect of age on the associations between genetically predicted PP and risk of ischemic stroke (*P*=0.22), but there was a significant association only in the higher deciles (Figure 2A). No significant effects of age were identified on the associations of either genetically predicted MAP or PP with the risk of ICH, with higher genetically predicted MAP showing relatively consistent associations with higher risk across all age deciles, and genetically predicted PP showing no significant association (Figure 2B). These results were consistent when repeating the analyses in the UKB validation sample (sensitivity analysis, Figure S3).

**Figure 3.**
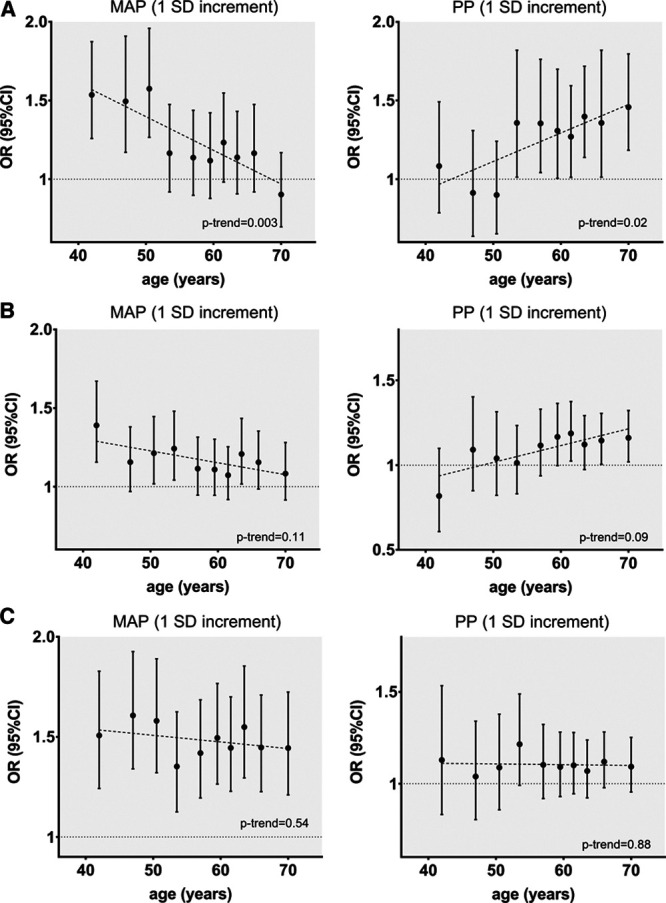
**Associations of genetically predicted mean arterial pressure (MAP) and pulse pressure (PP) across age deciles with risk of ischemic stroke etiological subtypes—(A) large artery stroke, (B) cardioembolic stroke, (C) small vessel stroke.** Effect sizes are derived from multivariable Mendelian randomization analyses adjusting for both genetically predicted MAP and PP in every decile. The analyses are based on the MEGASTROKE sample. Trends across age were explored with linear meta-regression analyses. OR indicates odds ratio.

**Figure 4.**
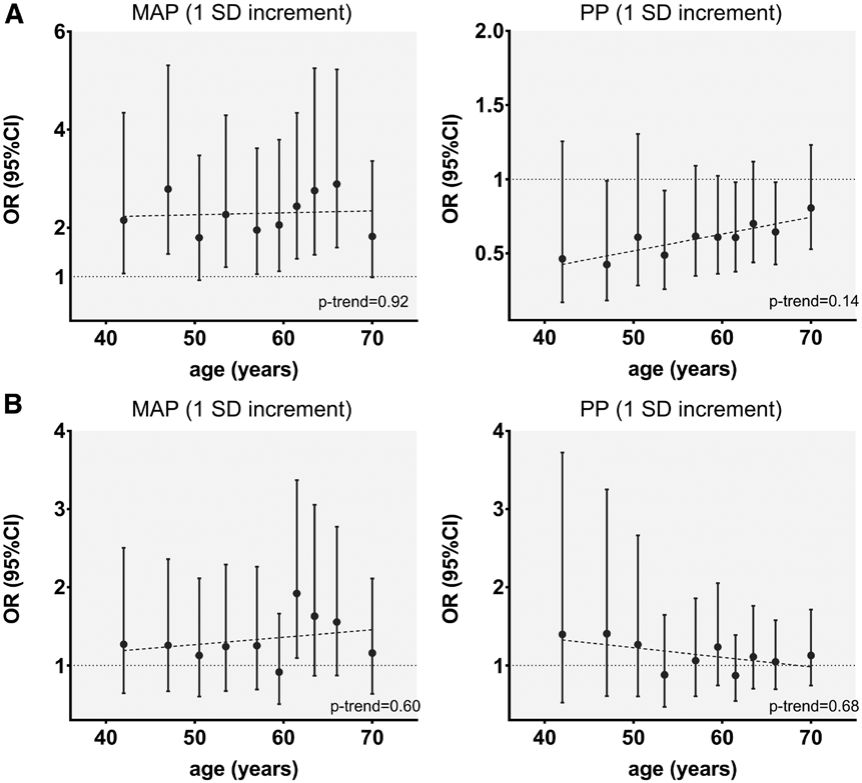
**Associations of genetically predicted mean arterial pressure (MAP) and pulse pressure (PP) across age deciles with risk of (A) deep and (B) lobar intracerebral hemorrhage.** Effect sizes are derived from multivariable Mendelian randomization analyses adjusting for both genetically predicted MAP and PP in every decile. The analyses are based on the ISGC sample. Trends across age were explored with linear meta-regression analyses. OR indicates odds ratio.

Next, we examined the effects of genetically predicted MAP and PP across the age deciles on ischemic stroke subtypes in MEGASTROKE and on location-specific ICH in ISGC. For large artery stroke, we found a significant attenuation of the effect of higher genetically predicted MAP with increasing age (*P*=0.003), as opposed to a gradually increasing effect of genetically predicted PP (*P*=0.02; Figure 3). For cardioembolic stroke, we identified no significant trends with increasing age, but only higher genetically predicted PP at older age deciles showed significant associations with higher risk while the opposite seemed true for MAP. For small vessel stroke, there was a uniform effect of higher genetically predicted MAP, but not PP, on higher risk across the entire age spectrum (Figure 3). For deep and lobar ICH, there were no trends for changing effects of genetically predicted MAP and PP across age (Figure 4). There were significant associations between higher genetically predicted MAP and higher risk of deep ICH, which seemed relatively consistent across age deciles. In contrast, there was no evidence of an association between either genetically predicted MAP or PP with lobar ICH at any age decile (Figure 4). Similar results were obtained in the dichotomous analyses for genetically predicted MAP and PP at age ≤55 and >55 years (Figure S4 and S5, Table S2).

## Discussion

In the current study, we employed MR to explore the independent effects of MAP and PP at different ages on risk of stroke. While genetically predicted MAP was associated with the risk of both ischemic and hemorrhagic across the entire examined age spectrum (38–71 years) independent of PP, its effect on ischemic stroke was attenuated with increasing age. On the contrary, genetically predicted PP showed an effect on ischemic stroke independent of MAP only at older ages. Across subtypes, this differential effect was mostly prominent for large artery stroke, for which we found genetically predicted MAP at younger age (≤55 years) and genetically predicted PP at older age (>55 years) to be associated with higher risk. Collectively, our findings highlight a causal role of late-life blood pressure pulsatility in ischemic stroke, in particular large artery stroke, on top of mean blood pressure.

It is still debated whether PP adds to cardiovascular risk stratification beyond SBP, DBP, and MAP.^34^ Current hypertension guidelines focus on SBP and DBP and do not typically consider PP,^35^ although the European guidelines, based on large-scale observational studies,^36,37^ acknowledge that a PP >60 mm Hg in older hypertensive individuals increases cardiovascular risk.^38^ Using MR, the current study extends previous evidence by supporting that widened PP in later life represents an additional causal risk factor for ischemic stroke on top of the well-established effects of mean blood pressure. Given the differential effects of antihypertensive drug classes on PP,^19,20,39^ our findings could have clinical implications for the optimal management of elevated blood pressure in later life. Indeed, our findings suggest that future clinical trials exploring the efficacy of blood pressure-lowering approaches for stroke prevention should also consider changes in PP. In addition, novel approaches specifically aiming at decreasing pulsatility in later life could offer benefit on top of current blood pressure-lowering strategies.

We found the effect of late-life PP to be particularly strong for large artery atherosclerotic stroke. This is in agreement with existing evidence suggesting an unfavorable effect of widened PP on atherogenesis, atheroprogression, and plaque instability.^40,41^ Interestingly, we found this effect to be augmented with increasing age, as opposed to an attenuating effect of increasing MAP. The precise mechanism of this finding remains unknown, but we assume the observed pattern to be the result of increasing arterial stiffness with age.^10,42^ Specifically, after an age of 50 to 60 years, the extracellular matrix of the arterial wall becomes less elastic, thus leading to decreased arterial compliance and increasing pulsatility.^42,43^

We found no evidence for an effect of genetically predicted PP at any age on risk of small vessel stroke or deep ICH, both of which are manifestations of cerebral small vessel disease.^44^ Previous observational studies on PP and neuroimaging markers of cerebral small vessel disease have provided inconsistent results.^45^ However, these results primarily originate from cross-sectional analyses of relatively small retrospective studies^45^ that are prone to confounding and reverse causation. On the contrary, we found the main determinant of both small vessel stroke and deep ICH throughout the examined age span to be MAP, in accord with the key role of elevated blood pressure on cerebral small vessel disease.^44^ Of note, we found no evidence for an association between either genetically predicted MAP or PP and lobar ICH. Unlike deep ICH, lobar ICH is related to cerebral amyloid angiopathy and the absence of an association signal between blood pressure and lobar ICH is consistent with observational^46^ and genetic^7^ data.

To our knowledge, our study represents the first attempt to employ MR for the exploration of temporal trends across the lifespan in the associations between a risk factor and an outcome. Traditional MR examines genetic predisposition to a phenotype at any timepoint and may be interpreted as the effects of lifelong exposure to this phenotype on the outcome of interest.^47^ By exploiting data from the UKB, we were sufficiently powered to explore the independent effects of genetically predicted MAP and PP on risk of stroke and its etiological subtypes across age deciles. We believe this approach could inform the design of future clinical trials including the optimal age group and the development of strategies for personalized medicine.

Our study also has limitations. First, the 2-sample MR setting might not be ideal for exploring temporal trends. For example, we cannot exclude that some of the stroke cases included in the outcome samples occurred at ages younger than the ages at which we examined the genetic determinants of blood pressure. Yet, most of the stroke cases in MEGASTROKE and ISGC occurred long after the cutoff of 55 years (mean age of cases 68.2 and 69.9 years, respectively), and our results were remarkably consistent in sensitivity analyses including only first-ever incident cases >55 years of age in the UKB that occurred after the baseline blood pressure measurements. However, this sensitivity analysis could not be done for the stroke subtypes, as information on subtypes was not available in the UKB. Second, the different genetic determinants of blood pressure across the examined age groups might in part reflect a higher probability of older individuals to receive antihypertensive medications. To account for this, we adjusted for the effects of antihypertensive medication in the original GWASs. Still, we cannot exclude a residual bias of this type on our findings. Third, as opposed to MAP, the variance explained by the genetic variants for PP was substantially lower at age ≤55 years as compared with >55 years. While this could have resulted in insufficient power to detect significant associations between PP at younger ages with the risk of stroke, this would be unlikely to explain the trend of the increasing association with large artery stroke in higher age deciles. Similarly, this would also be unlikely to result in the observed consistency of results in sensitivity analyses addressing the imbalance in the number of genetic instruments for the different phenotypes. Fourth, our analyses are based on brachial MAP and PP that were available in the UKB, which are less powerful predictors of future cardiovascular events as central measurements.^48^ Similarly, there were no 24-hour ambulatory blood pressure measurements in the UKB, which comprise the gold standard method of assessing blood pressure. Fifth, despite using the largest currently available GWAS data sets, our analyses for some of the stroke subtypes, especially for lobar and deep ICH subtypes may still have been underpowered. Finally, our analyses were primarily based on data sets involving individuals of European ancestry and might thus not be applicable to other ethnicities.

### Perspectives

Our study supports distinct effects of genetically predicted MAP and PP across the lifespan on stroke risk. While MAP seems to have a causal effect on both ischemic and hemorrhagic stroke across the entire age spectrum, we provide evidence for an additional independent effect of increasing PP at older ages on ischemic stroke, particularly large artery stroke. Our results complement findings from observational studies and warrant further investigation for the development of potential stroke preventive strategies targeting blood pressure pulsatility in later life.

## Acknowledgments

This research has been conducted using UK Biobank (UK Biobank application 2532), and summary data from the MEGASTROKE and the ISGC genome-wide association studies. M.K. Georgakis, D. Gill, R. Malik, A.J.S. Webb, and M. Dichgans designed the study. M.K. Georgakis and R. Malik performed the statistical analyses. All authors interpreted results. M.K. Georgakis, R. Malik, and M. Dichgans wrote the article. All authors edited the article for intellectual content. All authors take responsibility for the integrity of the study.

## Sources of Funding

M.K. Georgakis is funded by a scholarship from the Onassis Foundation. D. Gill was supported by the Wellcome Trust 4i Programme (203928/Z/16/Z) and British Heart Foundation Centre of Research Excellence (RE/18/4/34215) at Imperial College London. This project has received funding from the European Union’s Horizon 2020 research and innovation programme (666881), SVDs@target (to M. Dichgans; 667375), CoSTREAM (to M. Dichgans); the Deutsche Forschungsgemeinschaft as part of the Munich Cluster for Systems Neurology (EXC 2145 SyNergy—ID 390857198) and the CRC 1123 (B3; to M. Dichgans); the Corona Foundation (to M. Dichgans); the Fondation Leducq (Transatlantic Network of Excellence on the Pathogenesis of Small Vessel Disease of the Brain; to M. Dichgans); the e:Med program (e:AtheroSysMed; to M. Dichgans) and the FP7/2007-2103 European Union project CVgenes@target (grant agreement number Health-F2-2013-601456; to M. Dichgans).

## Disclosures

D. Gill is employed part-time by Novo Nordisk. The other authors report no conflicts.

## Supplementary Material



## Novelty and Significance

### What Is New?

- This is the first Mendelian randomization study exploring the independent causal effects of mean arterial pressure and pulse pressure on risk of stroke.
- This study further explores the differential effects of mean arterial pressure and pulse pressure across different age strata on risk of stroke and its subtypes.

### What Is Relevant?

- On top of the well-established effect of mean arterial pressure across the entire age spectrum on risk of ischemic and hemorrhagic stroke, this study provides evidence for an independent effect of genetically predicted pulse pressure in later life on risk of ischemic stroke.

### Summary

These findings provide evidence for a causal effect of late life blood pressure pulsatility on risk of ischemic stroke. Our results complement previous findings from observational studies and warrant further investigation for the development of potential stroke preventive strategies targeting blood pressure pulsatility in later life.
