# Supplementary figures and images for: Genetically Predicted Blood Pressure Across the Lifespan: Differential Effects of Mean and Pulse Pressure on Stroke Risk
Source: Hypertension. 2020 Jul 6;76(3):953–61. doi: 10.1161/HYPERTENSIONAHA.120.15136 (PMC7418931; doi:10.1161/HYPERTENSIONAHA.120.15136)

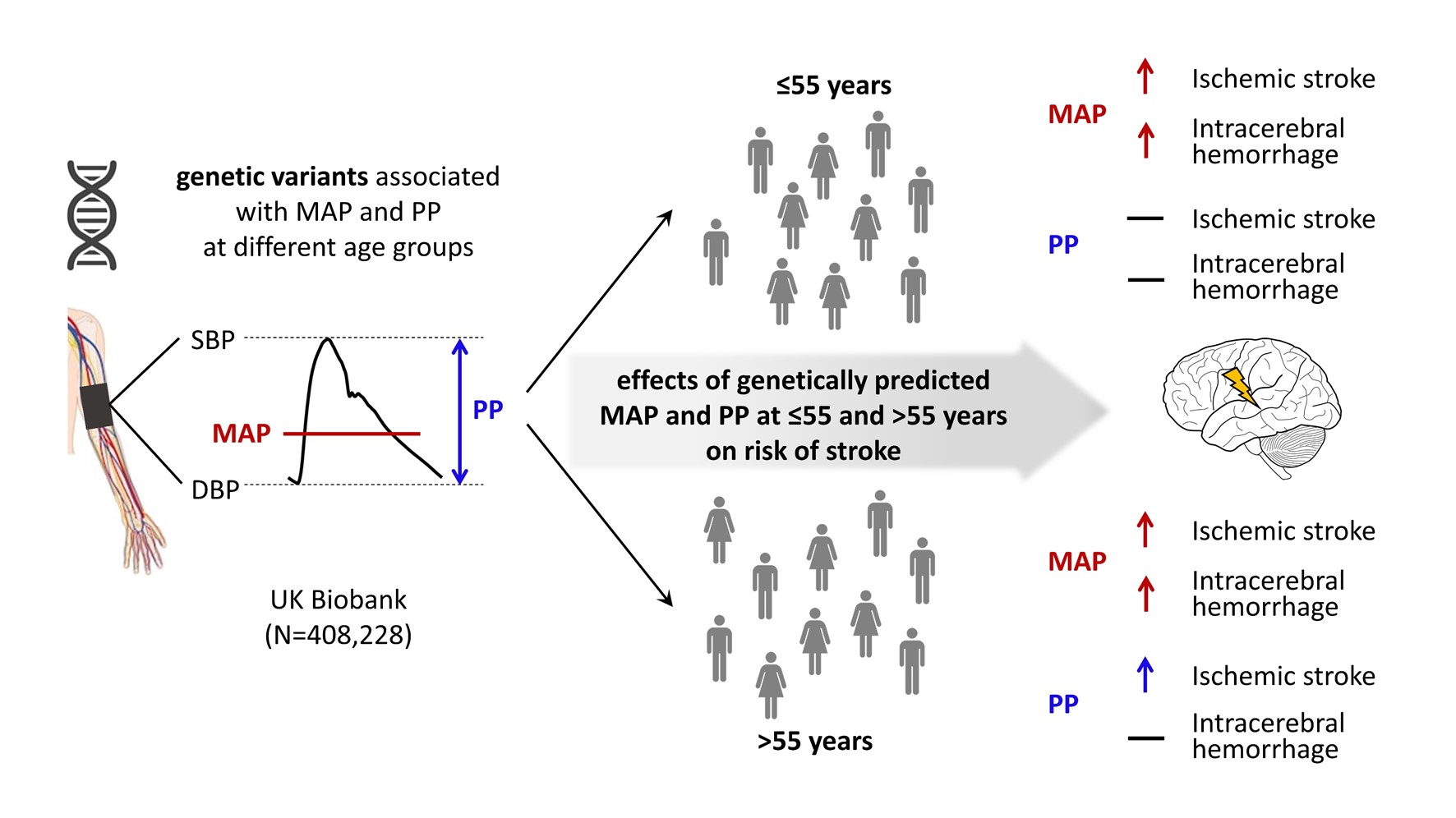

Supplement: Supplementary file 1 [file hyp-76-0953-s001.jpg]
