# Supplementary material for: Genetically Predicted Blood Pressure Across the Lifespan: Differential Effects of Mean and Pulse Pressure on Stroke Risk
Source: Hypertension. 2020 Jul 6;76(3):953–61. doi: 10.1161/HYPERTENSIONAHA.120.15136 (PMC7418931; doi:10.1161/HYPERTENSIONAHA.120.15136)
Supplement: Supplementary file 2 [file hyp-76-0953-s002.pdf]

# DATA SUPPLEMENT

## Genetically predicted blood pressure across the lifespan: differential effects of mean and pulse pressure on stroke risk

Marios K. Georgakis, MD, PhD[1*], Dipender Gill, MD, PhD[2*], Rainer Malik, PhD[1], Athanase D. Protogerou, MD, PhD[3], Alastair J. S. Webb DPhil[4], Martin Dichgans, MD[1,5,6]

[1] Institute for Stroke and Dementia Research, University Hospital, LMU Munich, Munich, Germany

[2] Department of Biostatistics and Epidemiology, School of Public Health, Imperial College London, London, United Kingdom

[3] Cardiovascular Prevention and Research Unit, Department of Pathophysiology, National and Kapodistrian University of Athens, Athens, Greece

[4] Centre for Prevention of Stroke and Dementia, Department of Clinical Neurosciences, University of Oxford, Oxford, United Kingdom

[5] Munich Cluster for Systems Neurology (SyNergy), Munich, Germany

[6] German Centre for Neurodegenerative Diseases (DZNE), Munich, Germany

* these authors contributed equally to the manuscript

## Supplementary Table S1. Genetic variants used as instruments in the analyses.

MAF: minimum allele frequency; beta.ypp: beta for the associations with pulse pressure at age <55 years; se.ypp: standard error for the associations with pulse pressure at age <55 years; pval.ypp: p-value for the associations with pulse pressure at age <55 years; r2_ypp: variance explained by the variant in pulse pressure at age <55 years; beta.ymap: beta for the associations with mean arterial pressure at age <55 years; se.ymap: standard error for the associations with mean arterial pressure at age <55 years; pval.ymap: p-value for the associations with mean arterial pressure at age <55 years; r2_ymap: variance explained by the variant in mean arterial pressure at age <55 years; beta.opp: beta for the associations with pulse pressure at age ≥55 years; se.opp: standard error for the associations with pulse pressure at age ≥55 years; pval.opp: p-value for the associations with pulse pressure at age ≥55 years; r2_opp: variance explained by the variant in pulse pressure at age ≥55 years; beta.omap: beta for the associations with mean arterial pressure at age ≥55 years; se.omap: standard error for the associations with mean arterial pressure at age ≥55 years; pval.omap: p-value for the associations with mean arterial pressure at age ≥55 years; r2_omap: variance explained by the variant in mean arterial pressure at age ≥55 years.

## Supplementary Table S2. Mendelian randomization associations of genetically determined mean arterial (MAP) and pulse pressure (PP) at age ≤55 and >55 years with ischemic stroke, intracerebral hemorrhage, and their etiological subtypes.

IVW: inverse variance weighted; Con-Mix: contamination mixture; MVMR: multivariable Mendelian randomization adjusted for both MAP and PP; IS: ischemic stroke; IS_UKB: ischemic stroke in the replication UK Biobank sample; ICH: intracerebral hemorrhage; ICH_UKB: intracerebral hemorrhage in the replication UK Biobank sample; LAS: large artery stroke; CES: cardioembolic stroke; SVS: small vessel stroke; DICH: deep intracerebral hemorrhage; LICH: lobar intracerebral hemorrhage.

## Supplementary Table S3. Mendelian randomization associations of genetically determined mean arterial (MAP) and pulse pressure (PP) across age deciles with ischemic stroke, intracerebral hemorrhage, and their etiological subtypes.

The effect sizes are derived from multivariable Mendelian randomization (MVMR) analyses adjusting for both genetically determined MAP and PP. IS: ischemic stroke; ICH: intracerebral hemorrhage; LAS: large artery stroke; CES: cardioembolic stroke; SVS: small vessel stroke; DICH: deep intracerebral hemorrhage; LICH: lobar intracerebral hemorrhage.

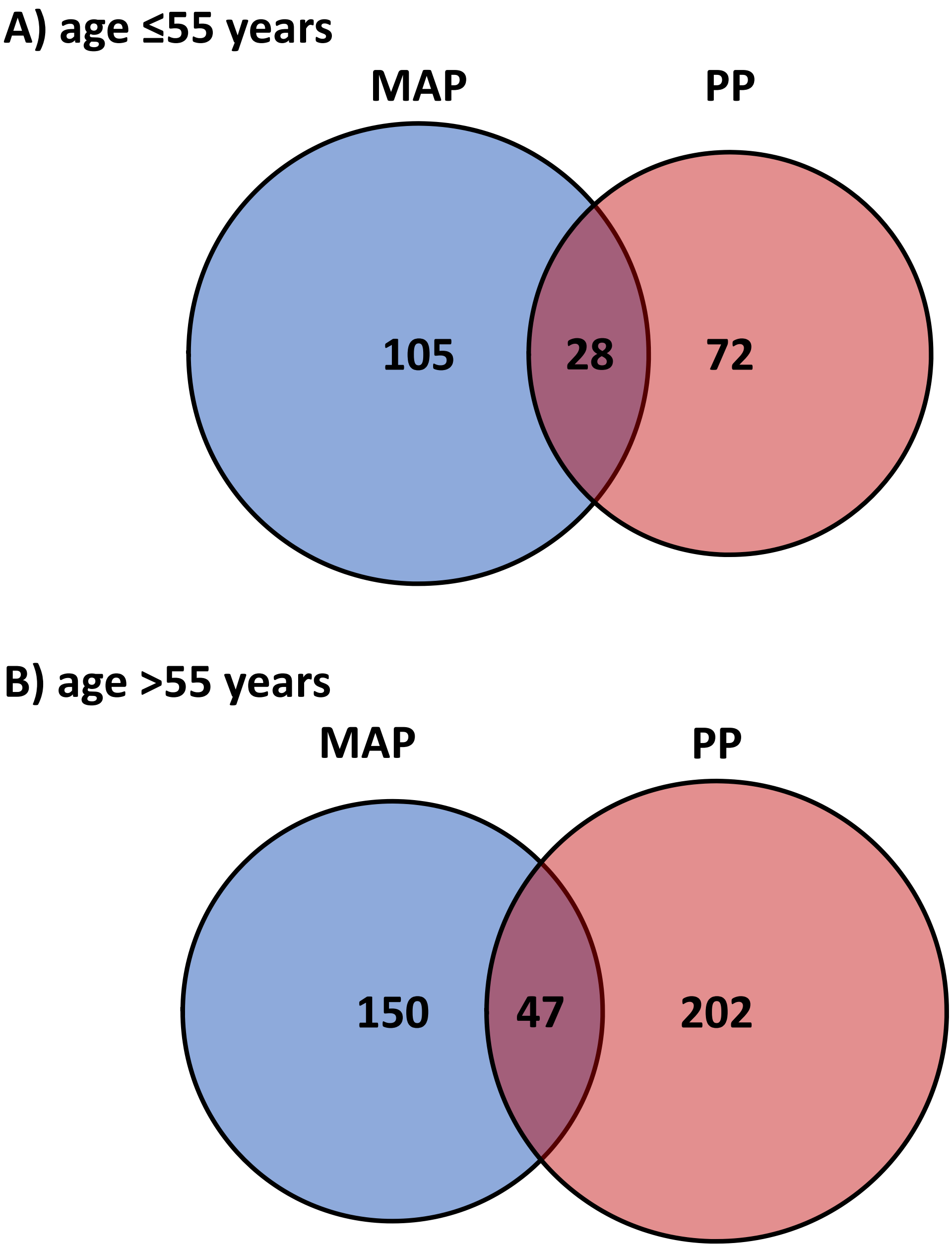


**Supplementary Figure S1. Overlap between genetic variants associated ($p<5x10^{-8}$) with mean arterial pressure (MAP) and pulse pressure (PP) at (A) age ≤55 and (B) >55 years that were used as instruments in this study.**

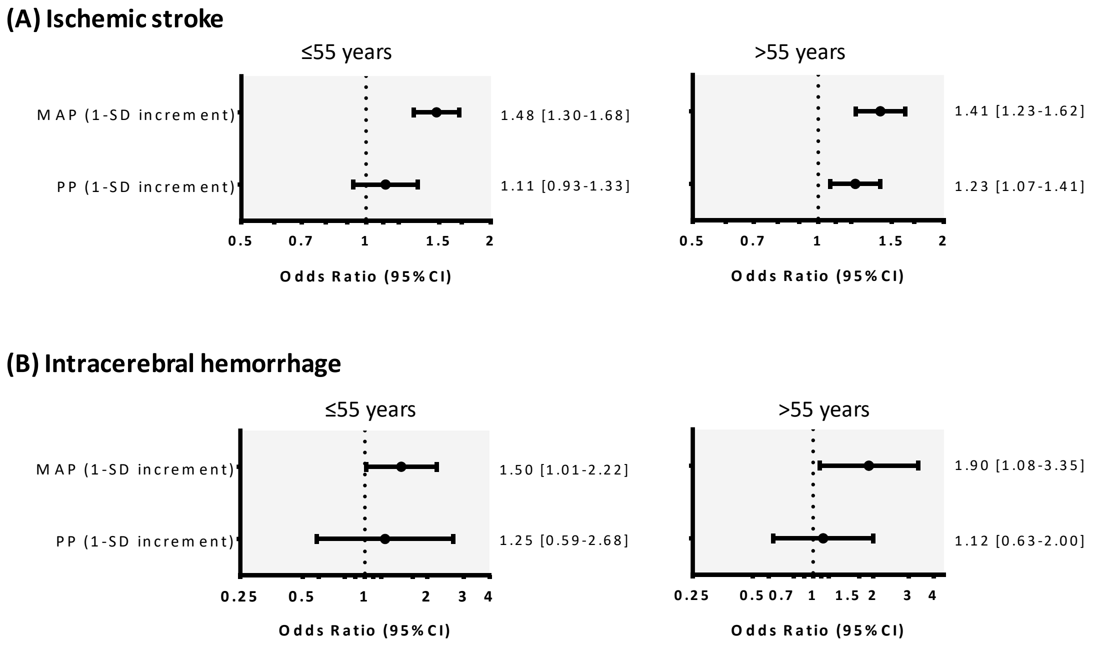


**Supplementary Figure S2. Associations of genetically determined mean arterial pressure (MAP) and pulse pressure (PP) at age ≤55 and >55 years of age with risk of ischemic stroke and intracerebral hemorrhage when randomly sampling 100 variants associated with MAP and 100 variants associated with PP.**

The Odds ratios correspond to the mean odds ratios obtained after randomly selecting as instruments 100 variants associated with MAP and 100 variants associated with PP and iterating 1000 times. Confidence intervals are derived based on the mean standard error of these analyses. This analysis aimed at attenuating the imbalance in the number of genetic instruments between MAP and PP. The effect sizes are derived from multivariable Mendelian randomization (MVMR) analyses adjusting for both genetically determined MAP and PP in MEGASTROKE.

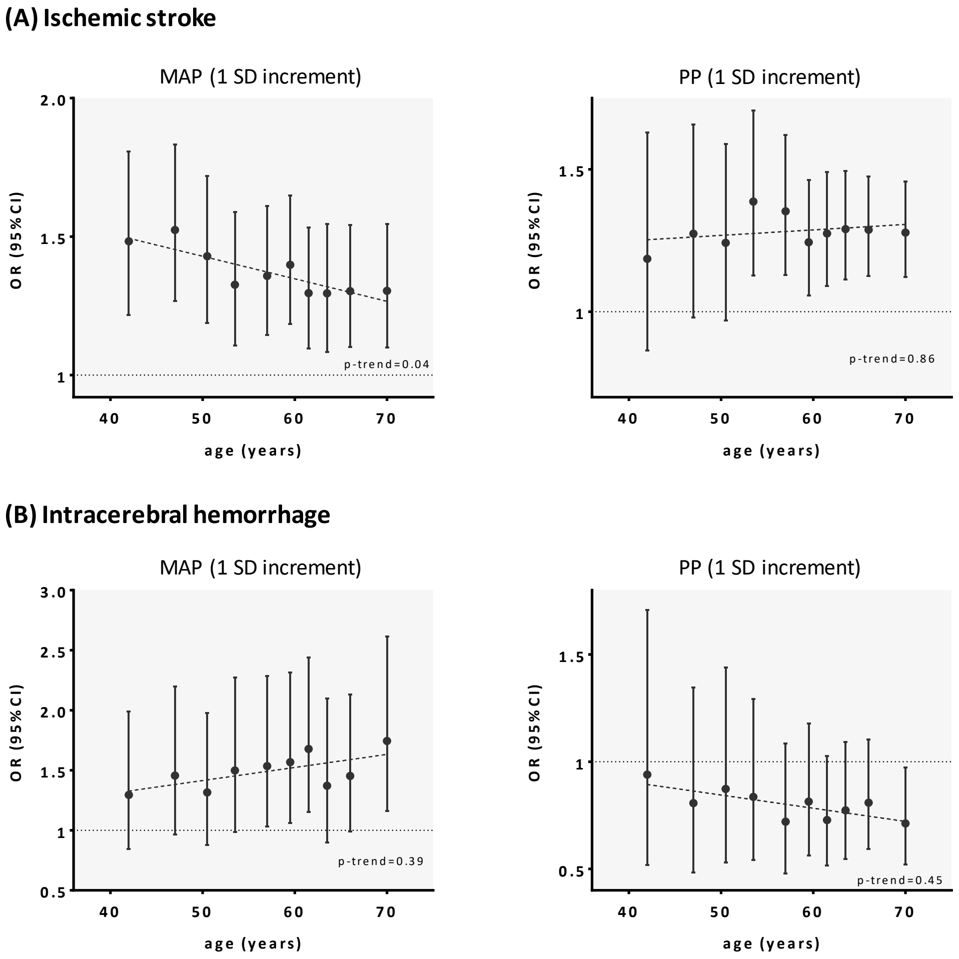


**Supplementary Figure S3. Associations of genetically determined mean arterial pressure (MAP) and pulse pressure (PP) across age deciles with risk of ischemic stroke and intracerebral hemorrhage in the UK Biobank.**

The effect sizes are derived from multivariable Mendelian randomization (MVMR) analyses adjusting for both genetically determined MAP and PP in every decile. The analyses are based on the UK Biobank and are restricted to incident ischemic stroke events that occurred after 55 years of age and after the blood pressure measurements. Trends across age were explored with linear meta-regression analyses.

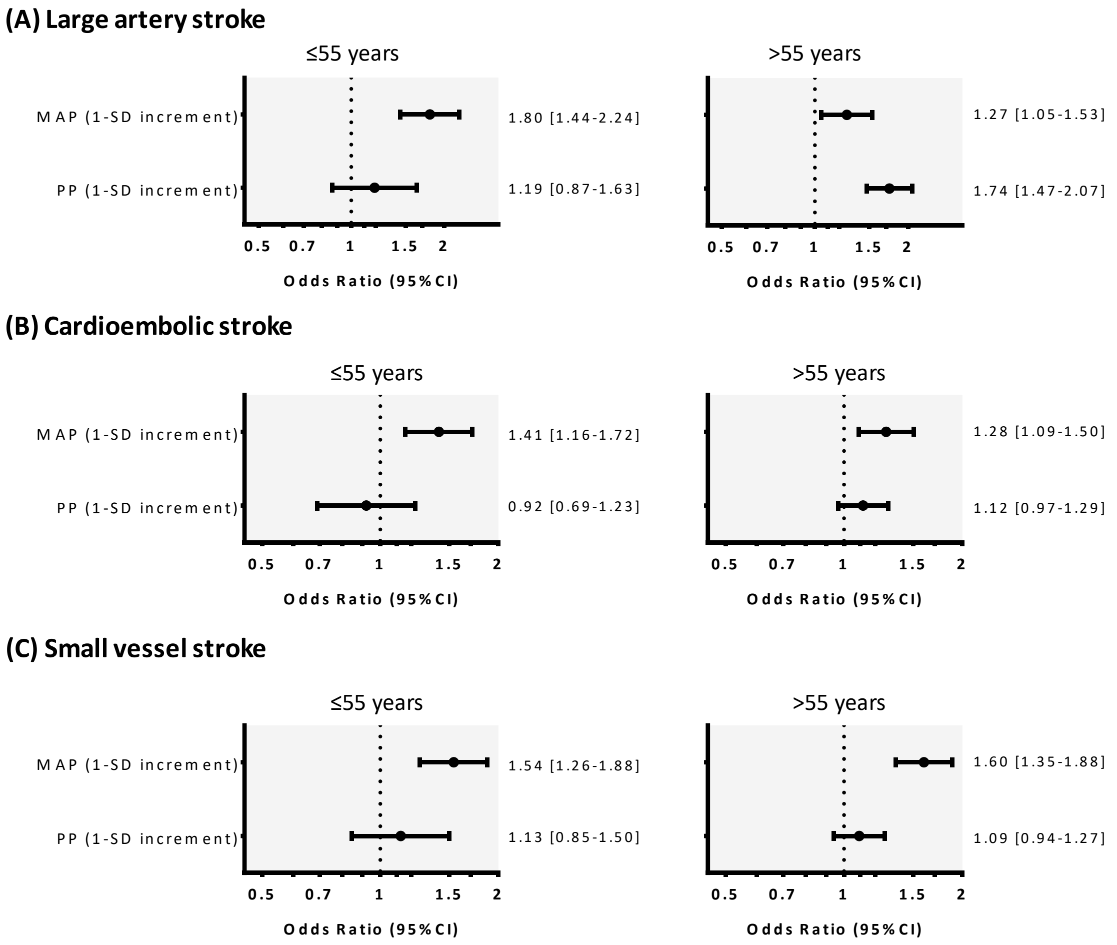


**Supplementary Figure S4. Associations of genetically determined mean arterial pressure (MAP) and pulse pressure (PP) at age ≤55 and >55 years of age with risk of ischemic stroke subtypes (large artery, cardioembolic, small vessel stroke).**

The effect sizes are derived from multivariable Mendelian randomization (MVMR) analyses adjusting for both genetically determined MAP and PP in MEGASTROKE.

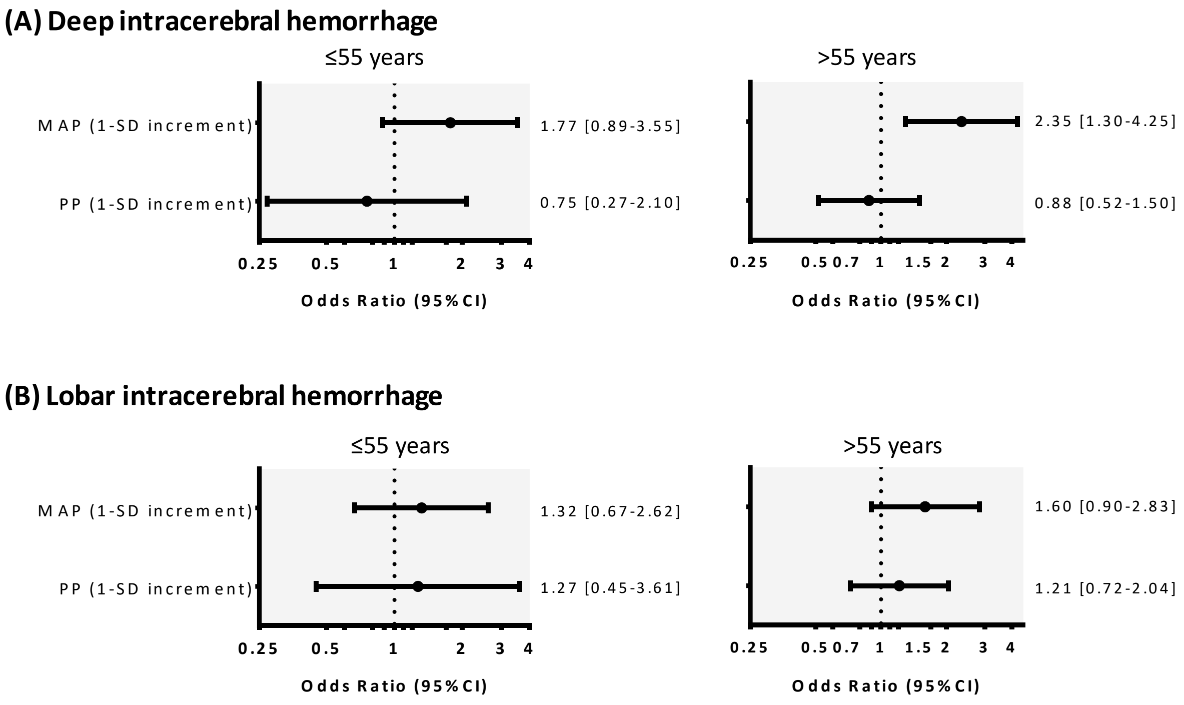


**Supplementary Figure S5. Associations of genetically determined mean arterial pressure (MAP) and pulse pressure (PP) at age ≤55 and >55 years with risk of deep and lobar intracerebral hemorrhage.**

The effect sizes are derived from multivariable Mendelian randomization (MVMR) analyses adjusting for both genetically determined MAP and PP in ISGC.
